# Maternal Primary Sjögren's Syndrome Complicated by Irreversible Fetal Third‐Degree Congenital Heart Block: A Case Report From Nepal

**DOI:** 10.1002/ccr3.71349

**Published:** 2025-10-24

**Authors:** Priety Shah, Pramit Jaiswal, Pukar Adhikari, Sumit Kumar Jaiswal, Kripasana Parajuli

**Affiliations:** ^1^ Department of Obstetrics and Gynecology Kathmandu Medical College and Teaching Hospital Kathmandu Nepal; ^2^ Department of Orthopedics Chitwan Medical College Bharatpur Nepal; ^3^ Himalayan Heart and Healing Bharatpur Nepal; ^4^ Nepalgunj Medical College Nepalgunj Nepal; ^5^ Kathmandu Medical College and Teaching Hospital Kathmandu Nepal

**Keywords:** antiLa/SSB, antiRo/SSA, congenital heart block, dexamethasone, fetal echocardiography, hydroxychloroquine, pregnancy, primary Sjögren's syndrome

## Abstract

In pregnancies of anti‐Ro/SSA– and anti‐La/SSB–positive women with primary Sjögren's syndrome, third‐degree fetal congenital heart block is usually irreversible despite corticosteroids. Preconception counseling, hydroxychloroquine prophylaxis, and serial fetal echocardiography from 16 weeks are essential to enable timely counseling, including discussion of termination in poor prognosis cases.

**Abbreviations**
β‐hCG: beta‐human chorionic gonadotropin (free β‐hCG)
11β‐HSD2: 11β‐hydroxysteroid dehydrogenase type 2
APLA: antiphospholipid antibodies
AV: atrioventricular
bpm: beats per minute
CHB: congenital heart block
EIA: enzyme immunoassay
G2A1: gravida 2, abortion 1
HCQ: hydroxychloroquine
IV: intravenous
LMP: last menstrual period
ms: milliseconds (for PR interval)
PAPP‐A: pregnancy‐associated plasma protein‐A
pSS: primary Sjögren's syndrome
SSA/Ro: Anti‐Sjögren's syndrome‐related antigen A
SSB/La: Anti‐Sjögren's syndrome‐related antigen B

## 1 Introduction

Primary Sjögren's syndrome (pSS) is a chronic autoimmune disease characterized by lymphocytic infiltration of exocrine glands, with systemic involvement and a strong female predominance in reproductive years [1]. Maternal anti‐Ro/SSA and anti‐La/SSB autoantibodies can cross the placenta and injure the fetal atrioventricular (AV) node, resulting in congenital heart block (CHB) [2, 3]. Among anti‐Ro/SSA–positive pregnancies, the incidence of CHB is approximately 1%–2%, increasing to 17%–18% after a previously affected pregnancy [4, 5]. Hydroxychloroquine (HCQ) appears to reduce recurrence risk [6]. Fluorinated corticosteroids (e.g., dexamethasone) may reverse incomplete block if initiated early, but an established third‐degree CHB is typically irreversible [7, 8]. We report a case of irreversible third‐degree fetal CHB in a woman with pSS, highlighting surveillance, counseling, and therapeutic considerations.

## 2 Case Presentation

### 2.1 Patient Information

A 34‐year‐old Nepalese woman (G2A1) with a two‐year history of pSS presented for antenatal care. She had no hypertension, diabetes, or thyroid disease. Past obstetric history included a missed abortion at 10 weeks for which suction and evacuation were done 1 year back during which she was under medication for Sjögren's syndrome. Family history of maternal hypertension; paternal hypertension and diabetes were present. She was a nonsmoker, nonalcoholic, with normal bowel/bladder habits.

Preconception, she had been counseled (January 2023) about risks of antibody‐mediated fetal AV block between 16 and 26 weeks, its unpredictable and rapid progression (first to third degree), and possible outcomes (hydrops if fetal heart rate < 50 bpm; postnatal pacemaker if 50–75 bpm). Fetal PR interval screening and steroid management strategies were discussed.

Prepregnancy HCQ dose was documented as 150 mg once daily, which was hiked to 300 mg after the anomaly scan was done. Low‐dose aspirin (150 mg) and routine supplements were used during pregnancy.

### 2.2 Timeline

- *Last menstrual period*: August 29, 2023 (estimated due date June 1, 2024).
- *October 31, 2023 (9^+1^ weeks of gestation)*: Ultrasound—viable intrauterine pregnancy; small posterior myometrial fibroid; minimal subchorionic hemorrhage.
- *November 22, 2023 (12^+5^ weeks of gestation)*: Nuchal translucency scan—normal.
- *November 25, 2023*: Dual marker screen—low risk for trisomy's 21/18/13; free βhCG: 1.4 ng/mL; PAPPA: 3.83 mIU/mL.
- *January 11, 2024*: Routine visit; anomaly screening planned; APLA negative.
- *January 14, 2024 (19^+5^ weeks of gestation)*: Anomaly scan—irregular fetal heart rate (62–126 bpm) with prolonged fetal PR interval (> 120 ms), consistent with evolving AV block. Multidisciplinary discussion (obstetrics, rheumatology, pediatric cardiology). HCQ increased to 300 mg daily; dexamethasone 6 mg IV daily started for 3 days; continued aspirin, iron, folic acid, calcium. In‐hospital monitoring included vitals, fetal heart rate, five‐point glucose profile, fetal movement counts, bleeding surveillance, and urine ketones 6 hourly.
- *15 January 2024 (≈20 weeks of gestation)*: Fetal echocardiography—complete (third‐degree) AV block with AV dissociation; atrial rate 130–140 bpm; ventricular rate 61–64 bpm. Oral dexamethasone 8 mg once daily commenced for 18 days. Ventricular function was preserved.
- *After 3 weeks (≈22 weeks of gestation)*: No improvement. Following counseling regarding poor fetal prognosis and risks, the couple opted for termination.

### 2.3 Diagnostic Assessment

**FIGURE 1:**
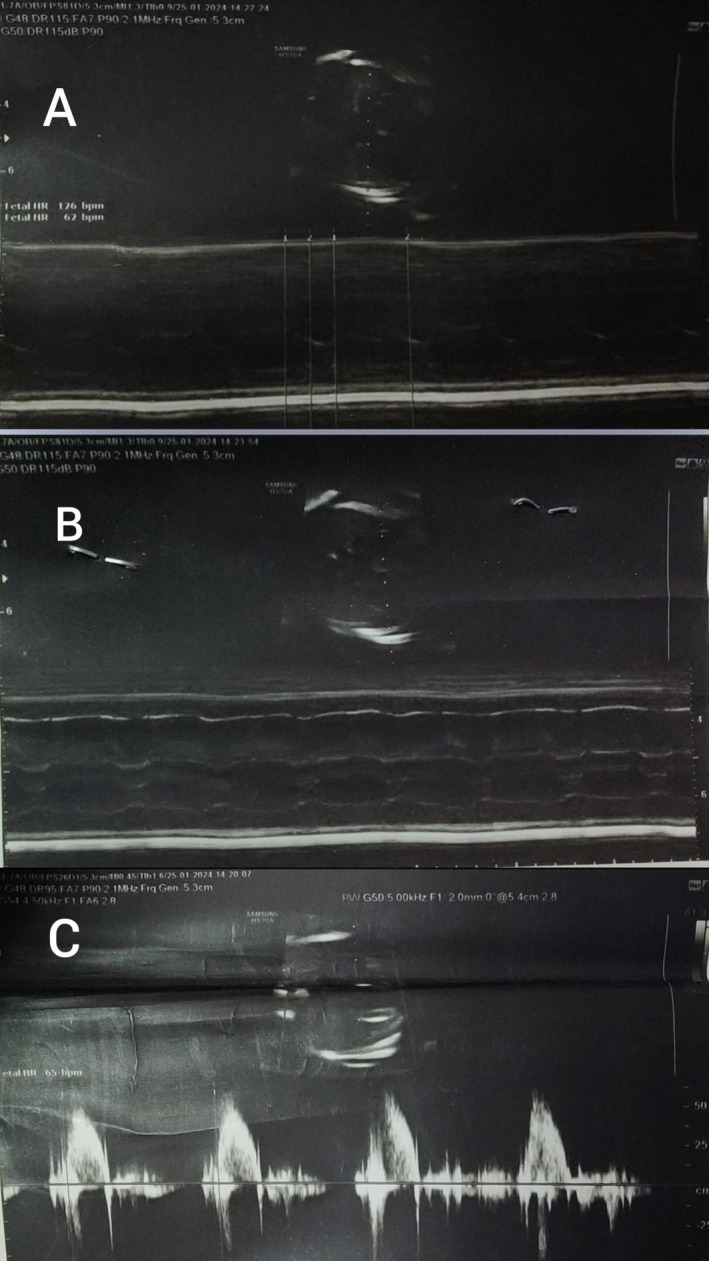
Ultrasound assessment of fetal cardiac activity. (A) M‐mode tracing showing variable fetal heart rate (FHR) ranging from 62 to 126 beats per minute (bpm). (B) M‐mode demonstrating fetal cardiac activity with bradycardia. (C) Pulsed‐wave Doppler tracing illustrating FHR of approximately 65 bpm.

**FIGURE 2:**
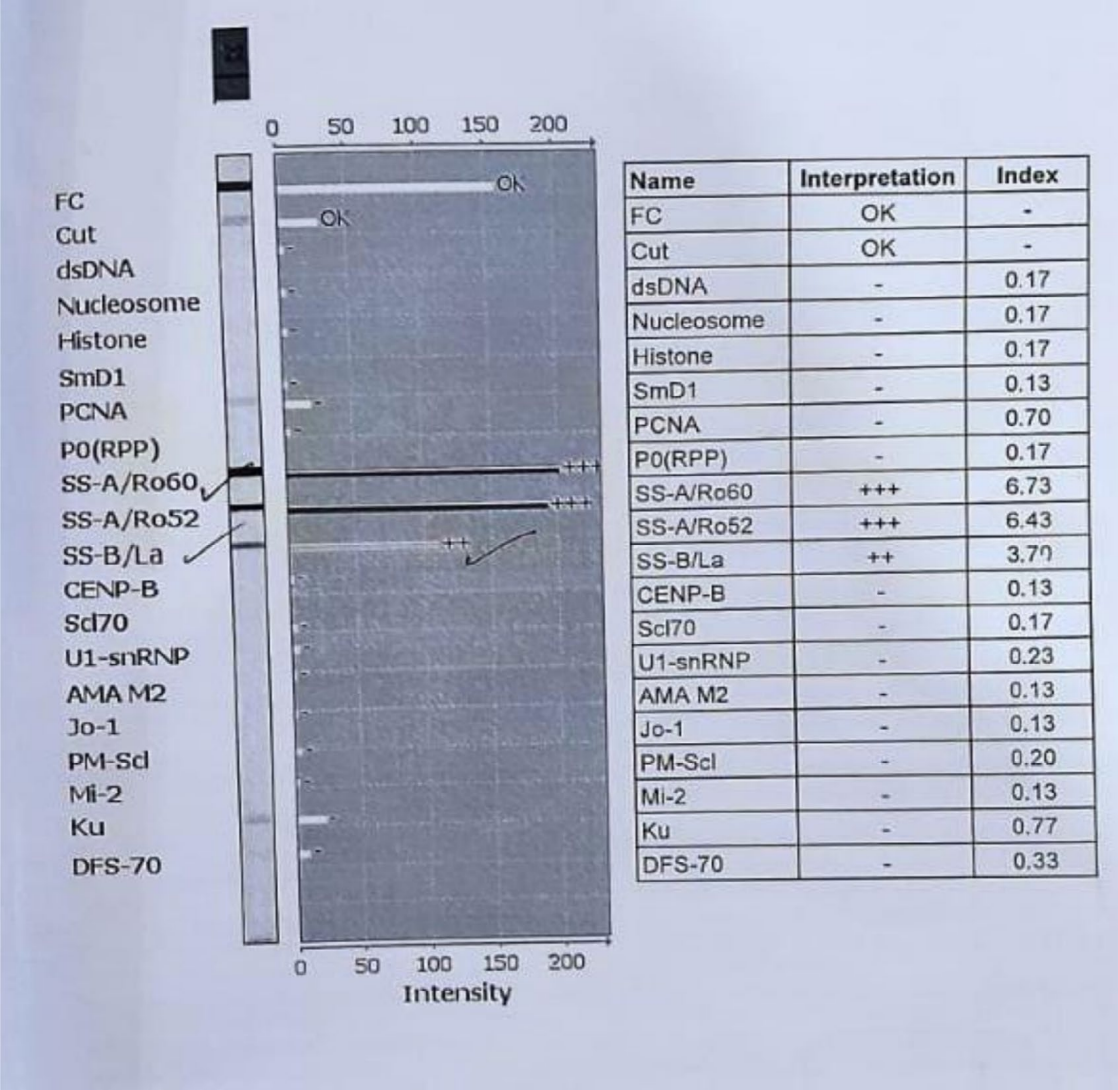
Maternal antibody profile showing strong anti‐Ro/SSA (Ro60 index 6.73; Ro52 index 6.43) and moderate anti‐La/SSB (index 3.70).

Findings of bradycardia, prolonged fetal PR interval on ultrasound, and AV dissociation on fetal echocardiography established the diagnosis of immune‐mediated CHB in the setting of maternal anti‐Ro/SSA and anti‐La/SSB antibodies. The initial anomaly scan at 19 + 5 weeks demonstrated an irregular fetal heart rate (62–126 bpm) with a prolonged PR interval (> 120 ms), suggestive of first‐ or second‐degree block, which rapidly progressed to confirmed third‐degree block on follow‐up echocardiography the next day, highlighting the swift evolution typical of this condition. Ultrasound assessment of fetal cardiac activity (Figure 1) shows M‐mode with variable fetal heart rate (FHR) ranging from 62 to 126 bpm, fetal bradycardia, and pulse wave Doppler tracing fetal heart rate of 65 bpm. She had sicca symptoms and strongly positive antibodies (Figure 2): anti‐Ro/SSA (Ro60 index 6.73; Ro52 index 6.43; EIA 118.87 units) and anti‐La/SSB (index 3.70; EIA 78.25 units). Antiphospholipid antibodies (anticardiolipin IgG/IgM, anti‐β2‐glycoprotein IgG/IgM) were negative on January 11, 2024. No structural anomalies were noted on first‐trimester scans.

### 2.4 Therapeutic Interventions

- *Baseline/maintenance*: HCQ (150 mg once daily early in gestation; increased to 300 mg twice daily on January 14, 2024; during hospital admission, aspirin 150 mg once daily, iron/folic acid, and calcium.
- *For evolving CHB*: Dexamethasone 6 mg IV once daily for 3 days (diluted and given slowly), then oral dexamethasone 8 mg once daily for 18 days.
- *Alternative agents such as salbutamol* (a β‐sympathomimetic) were not administered, as the ventricular rate remained stable (61–64 bpm) without hydrops, and evidence for its efficacy in established third‐degree block is limited.
- *Termination regimen (≈22 weeks)*: Intraamniotic digoxin for fetal demise, followed by mifepristone and sublingual misoprostol for induction.

### 2.5 Outcome and Follow‐Up

**FIGURE 3:**
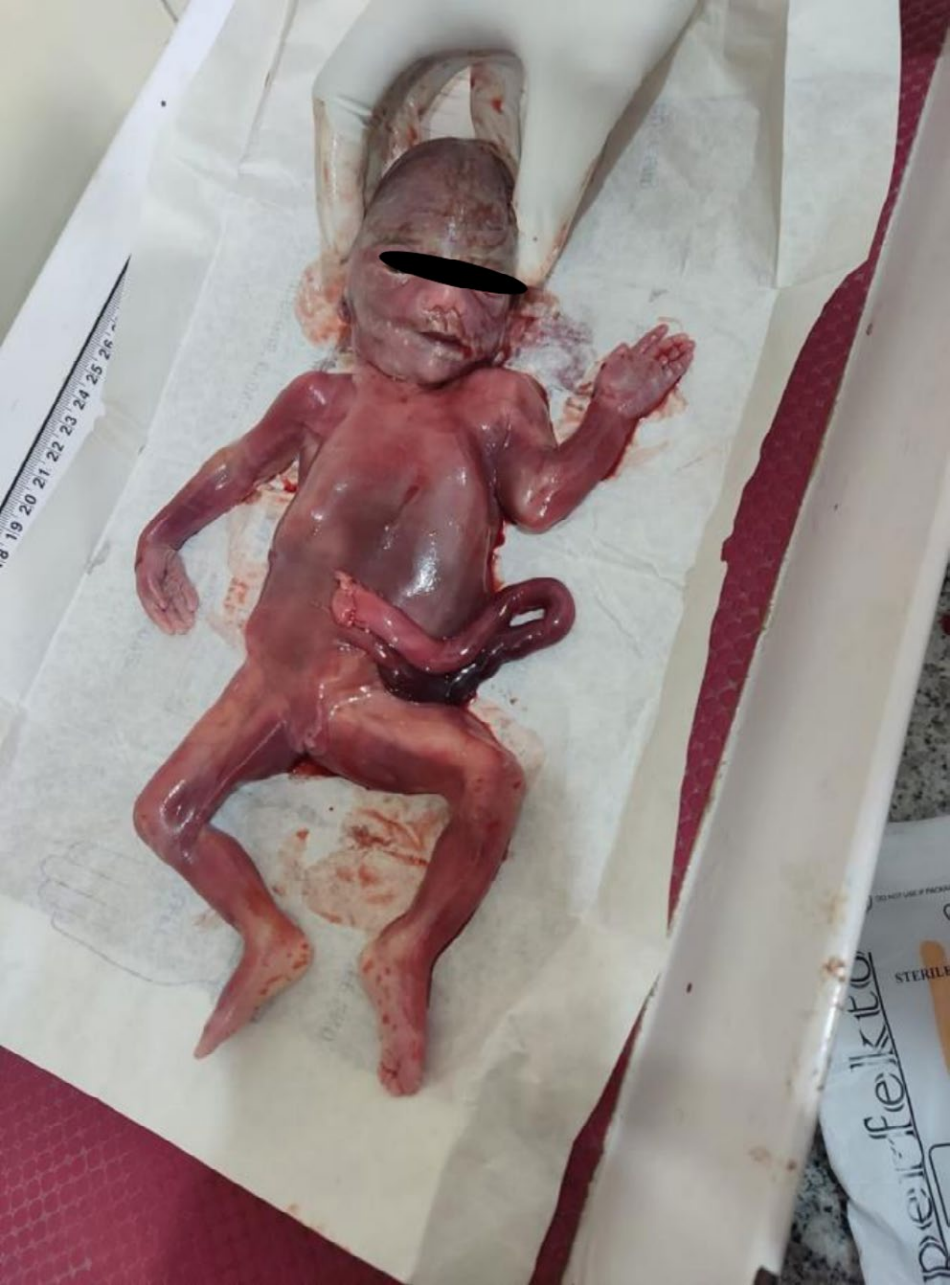
Post‐delivery image of the stillborn male fetus (1200 g), showing no gross congenital anomalies.

A stillborn male (Figure 3) was delivered at ~22 weeks with a birth weight of 1200 g, which was high for the gestational age. Gross external examination at delivery suggested no structural anomalies. No maternal complications from corticosteroids were recorded (e.g., hypertension, diabetes, or infection). She was discharged in stable condition with advice to continue HCQ and follow‐up with rheumatology and obstetrics for future pregnancy planning.

### 2.6 Patient Perspective

The patient and her husband expressed that early, clear counseling and coordination among specialists helped them make an informed, values‐concordant decision in a highly distressing situation.

## 3 Discussion

Autoimmune CHB results from transplacental passage of maternal IgG anti‐Ro/SSA and anti‐La/SSB antibodies that bind fetal conduction tissue, triggering inflammation, apoptosis, and subsequent fibrosis of the AV node [2, 3]. Once fibrosis and complete AV block are established, reversal is uncommon despite anti‐inflammatory therapy [8, 9]. Reported incidence among anti‐Ro/SSA–positive pregnancies is ~1%–2% (primary risk) with a markedly higher recurrence risk after an affected pregnancy [4, 5].

### 3.1 HCQ and Prevention

Observational and trial data suggest HCQ reduces recurrence of antibody‐mediated cardiac neonatal lupus/CHB and is recommended as prophylaxis in women with anti‐Ro/SSA antibodies, particularly with prior affected offspring [6]. In the multicenter prospective PATCH study, Izmirly et al. demonstrated that maternal HCQ at 400 mg daily significantly reduced the recurrence risk of CHB to 7.5% in anti‐Ro/SSA–positive women with a previously affected child, compared to historical rates of ~18% [10]. Similarly, Barsalou et al., in a systematic review and meta‐analysis, confirmed a protective effect against primary CHB (odds ratio 0.46), supporting its use even in first pregnancies with antibody positivity [11]. Despite these findings, HCQ is not expected to reverse established AV block, as illustrated in this case.

### 3.2 Corticosteroids and β‐Sympathomimetics

Dexamethasone readily crosses the placenta (unlike prednisolone, which is largely inactivated by placental 11βHSD2) [12, 13]. Several series report possible improvement when initiated at the earliest stages (first or some second‐degree block) [8, 9], whereas efficacy in third‐degree block is limited and inconsistent [8, 9, 14]. Alternative transplacental therapies, such as β‐sympathomimetics (e.g., salbutamol), have been explored to increase ventricular rate in cases with low rates and hydrops, as reported by Jaeggi et al. in a cohort where such agents improved outcomes in complete AV block without structural disease [15]. However, their use is debated due to insufficient randomized evidence, potential side effects (maternal tachycardia, fetal arrhythmia), and lack of endorsement in modern guidelines, favoring instead early detection and steroids for reversible blocks [8]. Potential maternal–fetal adverse effects (e.g., growth restriction and metabolic complications) necessitate judicious use of corticosteroids [16]. In our patient, dexamethasone was started when complete block had already developed, with no reversal.

### 3.3 Surveillance

Because progression from normal rhythm to complete block can occur within hours to days between 16 and 26 weeks, frequent fetal surveillance is advised (weekly or twice weekly PR interval/echocardiography in high‐risk pregnancies) to enable early detection and targeted intervention before fibrosis [8, 9]. Recent studies, such as Zhou et al., emphasize the value of serial Doppler echocardiography starting at 16 weeks in pSS pregnancies, reporting improved detection rates and outcomes with prompt intervention [12]. Our case was detected at 19 + 5 weeks, by which time complete heart block was present by 20 weeks.

### 3.4 Pregnancy and Outcome

Additionally, women with pSS face an elevated risk of adverse pregnancy outcomes, including spontaneous miscarriage. A recent meta‐analysis reported a relative risk of 8.85 for spontaneous abortion in SS pregnancies compared to controls [17]. Similarly, a multicenter study found that natural miscarriage was over 10 times more likely in pSS patients (12.80% vs. 1.52%; adjusted Odds ratio 11.335) [17, 18]. Updated analyses, such as Tan et al., reinforce these risks, attributing them to autoantibody‐mediated placental inflammation and advocating for optimized preconception management [18]. In this case, the patient's prior missed abortion at 10 weeks, which occurred after her pSS diagnosis, may have been related to her underlying autoimmune condition, underscoring the importance of preconception evaluation and management in such patients.

### 3.5 Counseling and Decision‐Making

Multidisciplinary counseling is essential to balance limited fetal therapeutic options against maternal risks and to discuss outcomes (hydrops risk when ventricular rate < 50 bpm; frequent postnatal pacemaker need when 50–75 bpm). In jurisdictions where permissible, termination may be an appropriate option in selected poor‐prognosis cases; our patient elected this after thorough counseling.

## 4 Conclusion

Established third‐degree immune‐mediated fetal CHB is rarely reversible. For anti‐Ro/SSA–positive pregnancies, preconception counseling, HCQ prophylaxis, and rigorous serial fetal echocardiography from 16 weeks are critical to detect early conduction changes when intervention has the greatest chance of benefit and to support informed, patient‐centered decisions, including pregnancy termination in select cases.

## Author Contributions

**Priety Shah:** conceptualization, data curation, investigation, project administration, supervision, writing – original draft, writing – review and editing. **Pramit Jaiswal:** conceptualization, data curation, investigation, project administration, writing – original draft, writing – review and editing. **Pukar Adhikari:** conceptualization, data curation, investigation, project administration, writing – original draft, writing – review and editing. **Sumit Kumar Jaiswal:** investigation, supervision, writing – original draft, writing – review and editing. **Kripasana Parajuli:** investigation, supervision, writing – original draft, writing – review and editing.

## Ethics Statement

Ethical approval was not required for a single‐patient case report under local policy. Institutional policies were followed.

## Consent

Written informed consent was obtained from the patient for publication of this case report and any accompanying images, consistent with journal policy. A copy is available for editorial review upon request.

## Conflicts of Interest

The authors declare no conflicts of interest.

## Supporting information


**Data S1:** CARE checklist for: Clinical Case report.

## Data Availability

No new datasets were generated. Deidentified clinical details are available from the corresponding author on reasonable request, in line with institutional policy.
