# Supplementary material for: Maternal Primary Sjögren's Syndrome Complicated by Irreversible Fetal Third‐Degree Congenital Heart Block: A Case Report From Nepal
Source: Clin Case Rep. 2025 Oct 24;13(11):e71349. doi: 10.1002/ccr3.71349 (PMC12550594; doi:10.1002/ccr3.71349)
Supplement: Supplementary file 1 — Data S1: CARE checklist for: Clinical Case report. [file CCR3-13-e71349-s001.docx]

**CARE Checklist for–: Clinical Case report**

| **Item** | **Topic** | **Checklist item description** | **Line/page** |
| --- | --- | --- | --- |
| 1 | **Title** | The words “case report” should be in the title along with the area of focus | √ |
| 2 | **Keywords** | Four to seven key words—include “case report” as one of the key words | √ |
| 3a | **Abstract** | **Background:** What does this case report add to the medical literature? | √ |
| 3b |  | **Case Presentation:** chief complaint, diagnoses, interventions, and outcomes | √ |
| 3c |  | **Conclusion:** What is the main “take-away” message from this case? | √ |
| 4 | **Introduction/background** | The current standard of care and contributions of this case—with references (1-2 paragraphs) | √ |
| 5 | **Timeline** | Information from this case report organized into a timeline (table or figure) | √ |
| 6a | **Patient Information** | De-identified demographic and other patient or client specific information | √ |
| 6b |  | Chief complaint—what prompted this visit? | √ |
| 6c |  | Relevant history including past interventions and outcomes | √ |
| 7 | **Physical Exam** | Relevant physical examination findings | √ |
| 8a | **Diagnostic** | Evaluations such as surveys, laboratory testing, imaging, etc. | √ |
| 8b | **Assessment** | Diagnostic reasoning including other diagnoses considered and challenges | √ |
| 8c |  | Consider tables or figures linking assessment, diagnoses and interventions | √ |
| 8d |  | Prognostic characteristics where applicable | √ |
| 9a | **Interventions** | Types such as life-style recommendations, treatments, medications, surgery | √ |
| 9b |  | Intervention administration such as dosage, frequency and duration | √ |
| 9c |  | Note changes in intervention with explanation | √ |
| 9d |  | Other concurrent interventions | √ |
| 10a | **Follow-up and** | Clinician assessment (and patient or client assessed outcomes when appropriate) | √ |
| 10b | **Outcomes** | Important follow-up diagnostic evaluations | √ |
| 10c |  | Assessment of intervention adherence and tolerability, including adverse events | √ |
| 11a | **Discussion** | Strengths and limitations in your approach to this case | √ |
| 11b |  | Specify how this case report informs practice or Clinical Practice Guidelines (CPG) | √ |
| 11c |  | How does this case report suggest a testable hypothesis? | √ |
| 11d |  | Conclusions and rationale | √ |
| 12 | **Patient Perspective** | When appropriate include the assessment of the patient or client on this episode of care | √ |
| 13 | **Informed Consent** | Informed consent from the person who is the subject of this case report | √ |
